# Potential of Metformin to Improve Cardiac Risk in Postpartum Women with Gestational Diabetes

**DOI:** 10.3389/fmed.2017.00180

**Published:** 2017-10-30

**Authors:** Oscar A. Viteri, Mary Alice Sallman, Pauline M. Berens, Pamela D. Berens, Farah H. Amro, Maria S. Hutchinson, Susan M. Ramin, Sean C. Blackwell, Jerrie S. Refuerzo, Judith. A. Smith

**Affiliations:** ^1^Department of Obstetrics, Gynecology and Reproductive Sciences, UTHealth McGovern Medical School, Houston, TX, United States; ^2^Department of Obstetrics and Gynecology, Baylor College of Medicine, Houston, TX, United States

**Keywords:** metformin, high-density lipoproteins, oxidized-low-density lipoproteins, gestational diabetes, cardiac risk, postpartum

## Abstract

**Objective:**

Pregnancy is associated with an increase in total cholesterol, high density lipoproteins (HDL), and low-density lipoproteins (LDL). Postpartum, HDL and LDL decrease over the first 12 weeks postpartum. Oxidized LDL (ox-LDL) is a marker of oxidative stress-related inflammation, which is associated with obesity and also with development of cardiovascular disease. Cardiovascular protection and weight loss are benefits from metformin, especially in women with diabetes. The objective of this study was to compare changes in lipid profiles and biomarkers for obesity during the initial 6 weeks postpartum between women with gestational diabetes mellitus (GDM) treated with metformin versus placebo.

**Methods:**

This was a planned ancillary study of a randomized controlled trial compares metformin versus placebo in women with GDM for postpartum weight loss. Two 3 mL blood samples were collected within 24 h of delivery and 6 weeks postpartum immediately processed after collection then stored at −20°C until completion of clinical trial prior to analysis. Change in the median plasma concentrations of total cholesterol, HDL, ox-LDL, glucose, insulin, leptin, and unacylated ghrelin were compared between study groups.

**Results:**

Of the 77 postpartum women were included, 35 received metformin and 42 received placebo. There was less of a reduction in HDL in the metformin group compared to placebo (−2.3 versus −7.5 mg/dL, *p* = 0.019). In addition, there was a greater reduction in ox-LDL in those receiving metformin (−12.2 versus −3.8 mg/dL, *p* = 0.038). No other differences were observed in the selected biomarkers evaluated.

**Conclusion:**

Biomarker levels of HDL and ox-LDL were positively affected during the initial 6 weeks postpartum in GDM women treated with metformin. Additional studies with a longer duration of metformin treatment in the postpartum period are warranted to evaluate long-term potential benefits.

## Introduction

The obesity epidemic is a major health-care problem worldwide. When in conjunction with insulin resistance and pancreatic β-cell dysfunction, obesity predisposes patients to type 2 diabetes mellitus (T2DM) later in life (1). In addition, obesity is associated with increased oxidative stress and inflammation, both of which are associated and can be predictors of cardiovascular disease (CVD) (2).

Obese pregnant women are predisposed to increased insulin resistance, excessive gestational weight gain, and inadequate postpartum weight loss resulting in increased abdominal adiposity and heightened risk of CVD (3, 4). Moreover, women with gestational diabetes mellitus (GDM) have a 50% lifetime risk of developing T2DM, further increasing their CVD risk (5, 6).

Metformin is a biguanide agent that decreases the hepatic production of glucose and the intestinal absorption of glucose. Treatment with metformin improves insulin sensitivity and overall improves carbohydrate tolerance to enhance weight loss, which indirectly reduces total cholesterol as well (7). More recently metformin is also being used as an alternative to insulin for the treatment of GDM. Studies have shown that metformin is likely safe and does not increase adverse pregnancy outcomes when used to treat GDM (8).

There are multiple biomarkers that can contribute to obesity pathways in pregnant women. Often the focus is on glucose, insulin, total cholesterol, high-density lipoprotein (HDL), and low-density lipoprotein (LDL) that are typically elevated in obese patients. Recent obesity research is focusing on other biomarkers that indirectly contribute to obesity and other related comorbidities such as CVC. These include: oxidized low-density lipoprotein [Oxidized LDL (ox-LDL)], leptin, and unacylated ghrelin. Ox-LDL is a marker of oxidative stress that associated with a higher risk of metabolic disease and CVD development; of note, ox-LDL is not related to the total cholesterol levels or HDL/LDL ratio (9). Elevations in ox-LDL would increase risk of CVD whereas lowering ox-LDL would decrease the risk of CVD. Ghrelin is a hormone that stimulates food intake and is associated with weight gain, while leptin is a hormone that suppresses appetite and is associated with weight loss. Leptin is secreted in proportion to levels of adipose tissue, and high levels of leptin are associated with oxidative stress (10). The purpose of this planned ancillary study was to evaluate the changes in lipid profiles and these selected biomarkers in postpartum women with GDM who received metformin.

## Materials and Methods

This was a planned ancillary study of a double-blinded, randomized clinical trial of metformin versus placebo for weight loss in puerperal women with GDM (11). This study was carried out in accordance with the recommendations of institutional review board (IRB) of the University of Texas Health Science Center at Houston and Children’s Memorial Hermann Hospital, Houston, TX, USA with written informed consent from all subjects. All subjects gave written informed consent in accordance with the Declaration of Helsinki. The clinical study with planned ancillary study was approved by the IRB of the University of Texas Health Science Center at Houston and Children’s Memorial Hermann Hospital, Houston, TX, USA. In brief, postpartum women diagnosed with GDM at ≥24 weeks’ gestation based on a 1-h glucose challenge test >200 mg/dL or by a confirmatory 3-h glucose tolerance test, and who delivered ≥34 weeks’ gestation were randomized to either metformin (850 mg daily for 7 days, the 850 mg twice daily for 5 weeks) or an equivalent placebo. The primary outcome of postpartum weight loss was similar between the study groups.

For the present study, non-fasting 3 mL blood samples of 77 puerperal women with GDM were collected within 24 h of delivery and at 6 weeks postpartum, immediately processed after collection, then stored at −20°C until completion of trial so that all immunoassays could be conducted at one time. These samples were then analyzed with immunoassays for glucose, total cholesterol, insulin, leptin, unacylated ghrelin, HDL, and ox-LDL. The Glucose Colorimetric Assay Kit was No. 10009582 was from Cayman Chemical (Ann Arbor, MI, USA) and the Total Cholesterol Assay Kit (Fluorometric) was No. STA-390 was from Cell Biolabs, Inc. (San Diego, CA, USA). The rat insulin, human leptin, and human unacylated ghrelin enzyme immunoassay kits were all purchased from Bertin Pharma and developed by Signosis, Inc. (Sunnyvale, CA, USA). The HDL and LDL/VDL quantification colorimetric/fluorometric kit was from BioVision Incorporated (Atlanta, GA, USA). All assays were performed in triplicate and according to the manufacturers’ protocols. The HT Synergy Multi-Mode Microplate Reader (BioTek, Winooski, VT, USA) was used to evaluate the absorbance for each respective assay. The insulin, leptin, and ox-LDL plate absorbance were all read at 450 nm and the glucose and unacylated ghrelin plate absorbance were read at 540 and 405 nm, respectively. The total cholesterol plate fluorescence was read with 590 nm excitation and 645 nm emission and the HDL plate O.D. was read at 562 nm. A change in the median plasma concentrations of total cholesterol, HDL, oxidized-LDL, glucose, insulin, leptin, and unacylated ghrelin was compared between groups using the Mann–Whitney *U-*test. Statistical analysis was conducted using STATA version 21. A *p*-value less than 0.05 was considered statistically significant.

## Results

**Figure 1:**
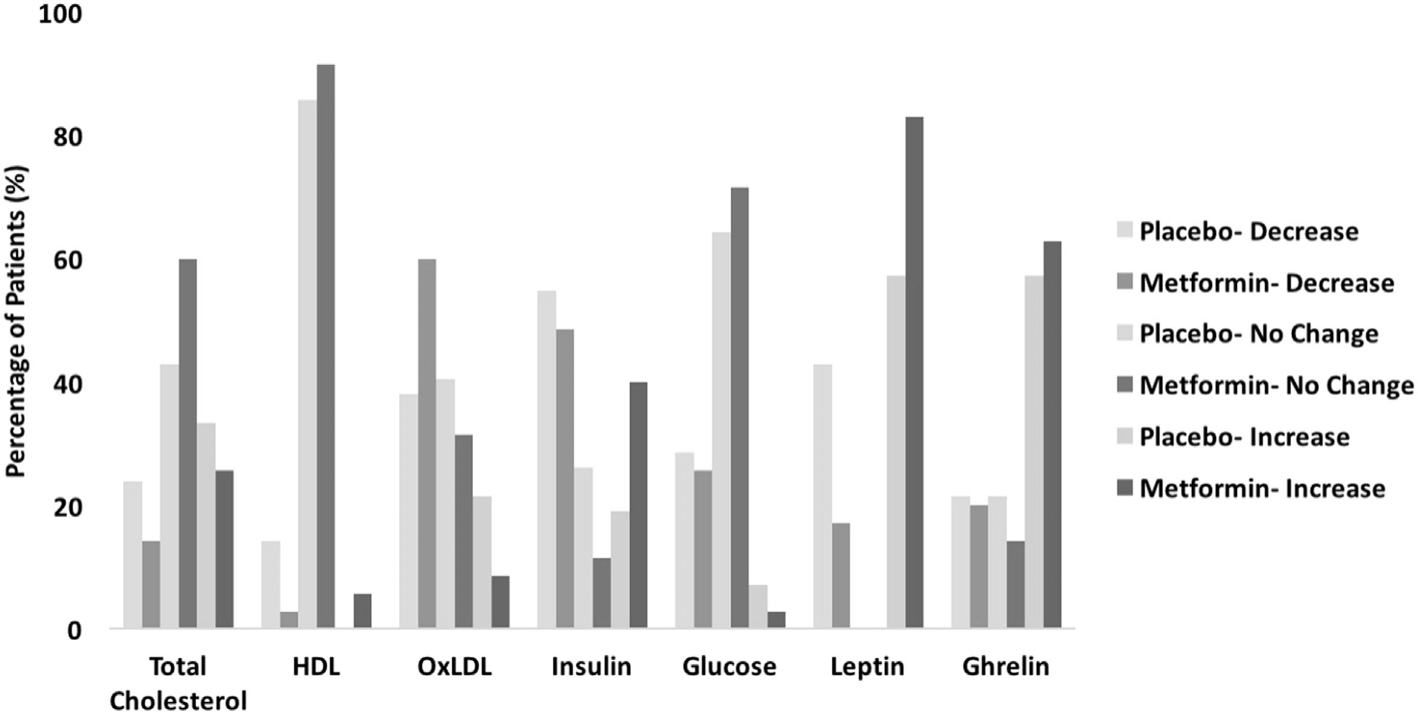
Summary of discernible biomarker level changes.

Of the total 77 postpartum women included in this ancillary study 35 were randomized to the metformin group and 42 to the placebo group. There were no differences noted in baseline clinical characteristics between the two study groups (11). All women were obese (i.e., body mass index ≥30 kg/m^2^) at the time of randomization. Although HDL decreased in both study groups after the initial 6 weeks postpartum, the magnitude of this decrease was greater in women who received placebo (median change of −7.5 mg/dL) compared to those receiving metformin (median change of −2.3 mg/dL), *p* = 0.019 (Table 1). In contrast, there was a larger decrease in ox-LDL levels observed in the metformin group (median change of −12.2 U/L) versus placebo group (median change of −3.8 U/L) *p* = 0.038. There was no significant difference in total cholesterol, glucose, insulin, unacylated ghrelin, and leptin between groups (Table 1). However, there was a favorable trend to improvement in all biomarkers except for ghrelin (Figure 1).

**Table 1:** Change in biomarker plasma concentrations.

	Metformin (*N* = 35)	Placebo (*N* = 42)	*p*-value
Total cholesterol	12.6 (−114 to 175)	17.1 (−294 to 143)	0.762
High-density lipoproteins	−2.3 (−25 to 39)	−7.5 (−40 to 14)	0.019
Oxidized low-density lipoproteins	−12.2 (−70 to 33)	−3.8 (−53 to 71)	0.038
Glucose	−9.2 (−85 to 58)	−4.2 (−50 to 33)	0.847
Insulin	1.8 (−850 to 1,382)	−100.2 (−910 to 343)	0.075
Unacylated Ghrelin	50.6 (−156 to 1,437)	51.8 (−202 to 650)	0.299
Leptin	12.4 (−17 to 33)	9.25 (−14 to 83)	0.339

*Data presented as median (range)*.

## Discussion

In this ancillary study of puerperal women with GDM, metformin improved metabolic biomarkers by (1) hindering the physiologic decrease in HDL levels and (2) accentuating the reduction in ox-LDL levels when compared to placebo. The lower levels of ox-LDL and slightly higher levels of HDL seen in women who received metformin suggest a decreased level of oxidative stress and silent inflammation during the study period compared to those who received placebo. A consistent decrease in oxidative stress and silent inflammation over time has been associated with promoting weight loss. In contrast, changes in total cholesterol, glucose, insulin, unacylated ghrelin, and leptin levels were equivalent between the groups.

High-density lipoproteins is a subcategory of total cholesterol. HDL represents about 20% of total plasma cholesterol and is inversely related to the occurrence of CVD, whereas ox-LDL is a marker of oxidative stress and is associated with a higher risk of metabolic disease and CVD development (9). It is plausible in this context that metformin may improve CVD risk by decreasing silent inflammation and steepening weight loss (12).

In women without GDM, HDL levels decrease during the initial 12 weeks postpartum, but the magnitude of this decline is small (13–15). Further, it has been shown that during pregnancy, women with GDM have lower levels of HDL than those without GDM (16).

The strengths of this ancillary study is that it is the first study to demonstrate potential benefit of metformin to also improve CVD risk by decreasing silent inflammation. Furthermore, these data suggest an additional mechanism, decreasing silent inflammation, in addition to its primary mechanism to facilitate weight loss by improving insulin sensitivity by reducing hepatic glucose production and decreasing intestinal glucose absorption. There are some limitations to this study that need to be acknowledged. Though significant changes were only seen in two biomarkers, significant fluctuations may perhaps be seen in the other biomarkers over a longer treatment period (greater than 6 weeks postpartum) given the favorable trend. While leptin has also been linked to risk of CVD, no significant change in leptin levels were observed in the samples from the women 6 weeks postpartum in this study. It is also interesting to note that no significant change in total cholesterol was seen in either the metformin or placebo group, though normally total cholesterol decreases postpartum. This could be due to the short time period of the study as well or to a difference in our patient population. Also, triglyceride levels were not recorded as the women were not fasting. As high serum triglycerides are known to be another risk factor for CVD, future studies should examine metformin’s effects on the level of this biomarker as well. This study also did not adequately account for the mothers’ breastfeeding status, though other studies have also shown that lipid levels can be affected by whether or not the mother was lactating (14). Further research should account for lactation status when evaluating the effects of metformin on lipids. However, this is the first study to analyze the potential short- and long-term effects of metformin on well characterized markers of metabolic dysregulation leading to CVD.

In conclusion, the metabolic biomarker results suggest that among postpartum women with GDM, metformin is superior to placebo in improving HDL and ox-LDL levels. Since pregnancy and the puerperium represent a window of opportunity to optimize maternal health, additional studies evaluating the effects of metformin on biomarkers for obesity and CVD in women with GDM over a longer period are warranted.

## Ethics Statement

This study was carried out in accordance with the recommendations of institutional review board (IRB) of the University of Texas Health Science Center at Houston and Children’s Memorial Hermann Hospital, Houston, TX, USA with written informed consent from all subjects. All subjects gave written informed consent in accordance with the Declaration of Helsinki. The clinical study with planned ancillary study was approved by the institutional review board (IRB) of the University of Texas Health Science Center at Houston and Children’s Memorial Hermann Hospital, Houston, TX, USA.

## Author Contributions

FA: patient accrual, sample acquisition, patient monitoring, manuscript preparation, and review. PDB, SB, and SR: study design, patient accrual, patient monitoring, manuscript preparation, and review. PMB and MS: sample analysis, data analysis and report, manuscript preparation, and review. MH and MS: patient randomization, sample processing and storage, manuscript preparation, and review. JR: clinical study principal investigator, study design, patient accrual, sample acquisition, patient monitoring, data monitoring, statistical analysis, manuscript preparation, and review. JS: laboratory studies principal investigator, laboratory study design/assay selections/validation, sample analysis, data analysis, interpretation and report, manuscript preparation and review, corresponding author.

## Conflict of Interest Statement

The authors declare that the research was conducted in the absence of any commercial or financial relationships that could be construed as a potential conflict of interest.
